# Allergic Rhinitis Improvement in Asthmatic Children After Using Acaricidal Bait: A Randomized, Double-Blind, Cross-Placebo Study

**DOI:** 10.3389/fped.2021.709139

**Published:** 2021-09-22

**Authors:** Ming Chen, Yufen Wu, Shuhua Yuan, Mingyu Tang, Lei Zhang, Jiande Chen, Luanluan Li, Jinhong Wu, Jing Zhang, Yong Yin

**Affiliations:** ^1^Department of Respiratory Medicine, Shanghai Children's Medical Center, Affiliated to Shanghai Jiao Tong University of Medicine, Shanghai, China; ^2^Department of Respiratory Medicine, Shanghai Children's Hospital, Affiliated to Shanghai Jiao Tong University, Shanghai, China

**Keywords:** allergic rhinitis, asthma, house dust mite, acaricidal bait, children

## Abstract

**Objective:** This study aimed to evaluate the effects of acaricidal bait use on the house dust mite (HDM) allergen concentration and occurrence of allergic rhinitis (AR) and asthma symptoms in children sensitized to HDMs.

**Study Design:** Sixty-six children (3–12 years old) with AR and asthma sensitized to HDMs were randomly assigned to receive an acaricidal bait intervention for 8 weeks and a placebo intervention for 8 weeks separated by a 4-week washout period. The visual analog scale (VAS) score, rhinitis control assessment test (RCAT) score, rhinoconjunctivitis quality of life questionnaire (RQLQ) score, asthma control questionnaire-5 (ACQ-5) score and HDM allergen levels were monitored.

**Results:** HDM allergen levels were significantly decreased after 8 weeks (Δder p2+f2 2.282 (3.516) μg/g vs. 0.147 (0.25) μg/g, *P* < 0.05) in the acaricidal bait group compared with the placebo group. The VAS, RCAT and RQLQ scores in the acaricidal bait group were also significantly improved (ΔVAS 7.5 (16) vs. 3 (18), *P* < 0.05; ΔRCAT−3 (5) vs. 0 (7), *P* < 0.05; ΔRQLQ 4.5 (8) vs. 1 (8), *P* < 0.05), but the ACQ-5 score did not improve (ΔACQ-5 0.2 (0.4) vs. 0 (0.65), *P* > 0.05).

**Conclusion:** Acaricidal bait reduced HDM exposure and improved rhinitis symptoms. This trial is registered at www.chictr.org.cn.

## Introduction

Allergic rhinitis (AR) is a non-infectious chronic inflammatory disease of the nasal mucosa that is primarily mediated by IgE after the body is exposed to allergens. AR is characterized by typical nasal itching, sneezing, watery secretions, and nasal mucosal congestion (1). Allergic asthma and AR are diseases that occur in different locations in the same airway, have similar pathological characteristics and exhibit chronic airway inflammatory responses. Epidemiological studies have shown that most asthma patients have rhinitis and that the presence of rhinitis is a risk factor for the development of asthma (2). Some children with allergic asthma combined with rhinitis have poor asthma control because they neglect the triggers of rhinitis (3). Asthma affects approximately 9.5% of pediatric patients, and AR affects approximately 8.5 to 14.6% of pediatric patients worldwide. The health cost burden and decline in quality of life have become health problems worldwide (4–7). Estimates show that 60-78% of patients who have asthma have coexisting AR (8). A Mayo Clinic study demonstrated that the use of beclomethasone topical nasal steroids in patients with ragweed allergic rhinitis and asthma can unexpectedly improve the symptoms of allergic rhinitis and asthma (9).

The occurrence and severity of allergy symptoms are related to exposure to environmental allergens. HDMs are common allergens that cause AR and asthma attacks in children (10). Avoiding allergen exposure is the basic principle in the treatment of allergic diseases. Reducing exposure to dust mites in the bedroom, especially in mattresses, is critical because mattresses are the main storage area of HDMs, and children spend most of their time in the bedroom since they sleep more than 8 hours a day. The current measures to reduce exposure to HDMs include keeping the room humidity below 50%, using HDM-impermeable bedding covers, removing carpets and stuffed toys, washing beddings frequently in hot water, and using high-efficiency particulate air filters or acaricides (11). A systematic review study (12) concluded that acaricides were the most promising measure used to reduce HDM exposure compared with the other abovementioned measures. Although traditional acaricides have a certain effect on killing mites, these pesticides do not penetrate carpets or mattresses and only kill live mites on surfaces. Most acaricides are also highly toxic. Their indoor use can cause irritation to human skin and eyes. Long-term repeated exposure is carcinogenic and teratogenic and poses other safety hazards.

The present study used a type of dust acaricide that is only slightly toxic (0.1% emamectin) and highly effective at attracting and killing dust mites. It induces dust mites to enter the acaricidal bag and then kills the dust mites to reduce allergens. This study aimed to investigate the effects of acaricidal bait on the daily clinical symptoms of AR and asthma in HDM-sensitized children and the level of HDM allergens in mattresses.

## Materials and Methods

### Study Design

**Figure 1:**
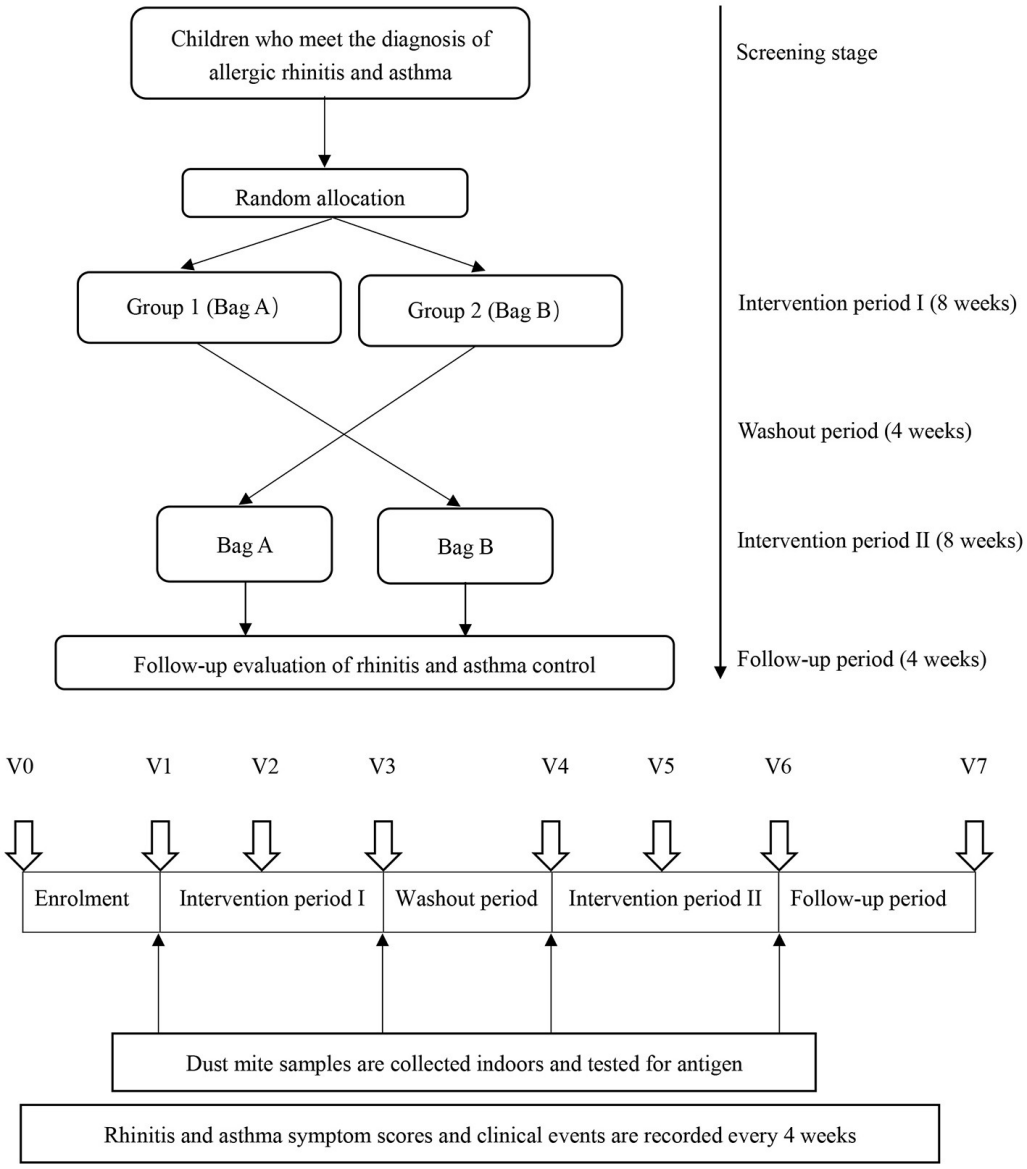
Study design. V0 = before the study began; V1 = the first day after enrolment; V2 = the fourth week plus or minus 3 days after enrolment; V3 = the eighth week plus or minus 3 days after enrolment; V4 = the twelfth week plus or minus 3 days after enrolment, V5 = the sixteenth week plus or minus 3 days after enrolment, V6 = the twentieth week plus or minus 3 days after enrolment; V7 = the twenty-fourth week plus or minus 3 days after enrolment.

This study was a randomized, double-blind, cross-placebo clinical trial. Before conducting the study, it was determined that at least 44 patients in each group were needed based on the assumption that a clinically relevant reduction of 25% (13) from a mean (±SD) score on the visual analog scale of 54 ± 27 (14) points could be detected with a power of 90% and a two-tailed alpha level of 0.05. Considering a 10% dropout rate, each group needed to enroll at least 49 patients. Sixty-six children were recruited for this placebo-controlled, double-blind crossover trial. The participants received the acaricidal bait intervention for 8 wks and the placebo intervention for 8 wks; the order of the interventions was random. A 4-wk washout period separated the two interventions. As a result, the placebo and acaricidal bait groups each included 66 patients. After registration, the participants provided basic information (V0 = before the study began), and qualified participants were randomly assigned. The acaricidal bait and placebo were identical in appearance and marked as bag A or bag B. Only pharmacists who were not involved in the clinical research knew the contents of bag A and bag B. Children with an odd and even number of random numbers were enrolled in Group 1 and Group 2, respectively. The trial process was explained, and informed consent was obtained before the start of the trial. Bag A and bag B were placed under the mattresses of the children in Group 1 and Group 2, respectively, for 8 wks. Then, the interventions were switched between the two groups for 8 wks after a 4-wk washout period. During the entire experiment, the staff sent questionnaires to the participants via WeChat to collect information regarding their AR symptoms, AR control, asthma control and adverse events every 4 wks (V1 = the first day after enrolment; V2 = the fourth week plus or minus 3 days after enrolment; V3 = the eighth week plus or minus 3 days after enrolment; V4 = the twelfth week plus or minus 3 days after enrolment; V5 = the sixteenth week plus or minus 3 days after enrolment; V6 = the twentieth week plus or minus 3 days after enrolment; V7 = the twenty-fourth week plus or minus 3 days after enrolment). At V1, V3, V4, and V6, the staff entered the room and used a glass fiber film vacuum cleaner to collect indoor samples from the mattresses (Figure 1). All participants, researchers, and physicians were blind to the allocations via the use of random codes until the statistical analyses were completed. During the 24-week research period, professional physicians answered the participants' questions. Compliance was monitored at each 4-week follow-up. Subjects who did not complete the questionnaires after 3 reminders were excluded from the study.

### Participants

#### Inclusion Criteria

1. Aged 3–12 years, male or female.
2. Children diagnosed with AR and asthma in accordance with the 2019 guidelines for the diagnosis and treatment of AR (15) and the diagnostic criteria for childhood asthma formulated by the National Children's Asthma Prevention and Treatment Cooperation Group in 2016 (16).
3. Maintained use of guide-based rhinitis and/or asthma control drugs for the past 1 month (Mometasone Furoate Aqueous Nasal Spray for children aged 3 and older; Budesonide Nasal Spray for children aged 6 and older; Fluticasone Propionate Nasal Spray for children aged 12 and older; Fluticasone Propionate Inhaled Aerosol for children aged 3 to 5 years; Salmeterol Xinafoate and Fluticasone Propionate Aerosol for children aged above 5 years; and Budesonide and Formoterol Fumarate Powder for Inhalation for children aged above 6 years).
4. Performance of a serum-specific allergen test, with the level of dust mite allergen sIgE > 0.35 IU/mL considered positive.
5. Informed consent signed by the guardians of all subjects.
6. Agreement to collect dust mites from indoor mattresses.

#### Exclusion Criteria

1. Basic diseases, such as congenital heart disease, immune deficiency, gastroesophageal reflux, bronchopulmonary dysplasia, and obliterative bronchiolitis.
2. Inability to sleep in a separate bed.
3. Participation in other clinical studies within the past 3 months.

### Intervention

During the intervention, all patients received interventions with acaricidal bait and placebo. The acaricidal bait and placebo had the same appearance. The main acaricidal ingredient in the dust mite package was 0.1% emamectin, and the placebo package contained high temperature-sterilized sand.

### Adverse Event Monitoring

During the trial, acaricidal bait was placed under the mattress and was not in direct contact with the child. However, the acute oral, transdermal, and inhalation toxicity of 0.1% emamectin in the mite bait was considered minimal. Some adverse events that may occur with emamectin include skin and eye irritation, nausea, vomiting, headache, dizziness, fatigue, chest tightness, excessive sweating, salivation, blurred vision, convulsions, and tachycardia or bradycardia.

The severity should be defined according to the following criteria:

Mild: Awareness of signs or symptoms but easily tolerated.
Moderate: Sufficient discomfort causing interference with normal daily activities.
Severe: Inability to perform normal daily activities.
Life threatening: Immediate risk of death from the reaction that occurs.

All adverse events were tracked until the incident was resolved or the study was completed.

### Endpoints

The primary endpoint was the VAS score, Patients were scored with a VAS score for symptoms occurring in the past week. A total of eight symptoms including sneezing, rhinorrhoea, nasal itching, nasal congestion, itchy eyes, teary eyes, foreign body sensation and red eyes using a 10-cm-long ruler, 0 ~ 10, to show the severity of the patient symptoms (“0” for no such symptoms and “10” representing the heaviest of such symptoms). Overall scores range from 0 (no symptoms) to 80 (very severe symptoms) (17). The secondary endpoints were the dust mite antigen concentrations in mattresses, RCAT, RQLQ, ACQ-5, and adverse events. RQLQ (18) is composed of 14 rhinitis problems assessed by children with rhinitis in the past 1-2 weeks in terms of their symptoms, mental state, psychological state, social interaction, etc. Each problem:0: normal;1: Slight;2 points: moderate;3: serious,4: very serious. The higher the score, the more serious the impact of rhinitis on quality of life. The RCAT (19) has 6 items that include nasal congestion, sneezing, watery eyes, sleep problems caused by rhinitis, activity avoidance, and rhinitis symptom control. Responses are measured on 5-point Likert-type scales. RCAT scores range from 6 to 30, with higher scores indicating better rhinitis control. ACQ-5 is a scale composed of 5 simple multiple choice questions. The child will be asked to evaluate the level of asthma control in the past 1 week. The lower the score is, the better the control level. The electronic VAS, RCAT, RQLQ, and ACQ-5 questionnaires were sent via WeChat to collect information from V1-V7. Indoor HDM antigen sampling was performed at V1, V3, V4, and V6. Three sampling points were randomly selected for each mattress, and each sampling point had a range of 30 cm^2^. Each sampling point was vacuumed 10 times repeatedly using a glass fiber membrane mite-clearing vacuum cleaner, and the bed area was recorded simultaneously. Dust on the glass fiber membrane in the vacuum cleaner was placed in a plastic bag and stored at −20°C to kill the HDMs. Allergens were extracted from the samples from each family after weighing. The enzyme-linked immunoassay analysis (ELISA) method (Indoor Biotechnologies, Charlottesville, VA, USA) was used to detect the dust mite antigens Der p2 (*Dermatophagoides pteronyssinus*) and Der f2 (*D. farinae*) in the extraction solution.

### Statistical Analysis

The mean substitution method was used to fill in missing values. An independent sample *t*-test was used for the comparisons of the continuous variables (i.e., measurement data) that conformed to a normal distribution between the two groups. Non-parametric tests were used for skewed distributions. Categorical variables (count data) are expressed as constituent ratios, and a chi-square test was used for comparisons between the groups. The ACQ is a composite measure that assesses the asthma condition according to 5 items. An ACQ score of 0.75 or more was considered indicative of incompletely controlled asthma. According to the ACQ-5 scores, the subjects were divided into the well-controlled group and the incompletely controlled group. An RCAT score greater than 21 points was considered good control, and an RCAT score less than or equal to 21 points was considered incomplete control. A multivariate logistic regression model was used to evaluate the relative associated relationships between the binary outcome (ACQ-5 score ≥ 0.75) and a set of covariates. The odds ratios (ORs) and 95% CIs were estimated. Variables with *P*-values less than 0.05 in the univariate analysis were included in this model. The data are expressed as the means and SDs. A *P*-value less than 0.05 was considered statistically significant. SPSS 20.0 software (Chicago, IL, USA) was used to analyse the experimental data.

## Results

### Patient Population

**Figure 2:**
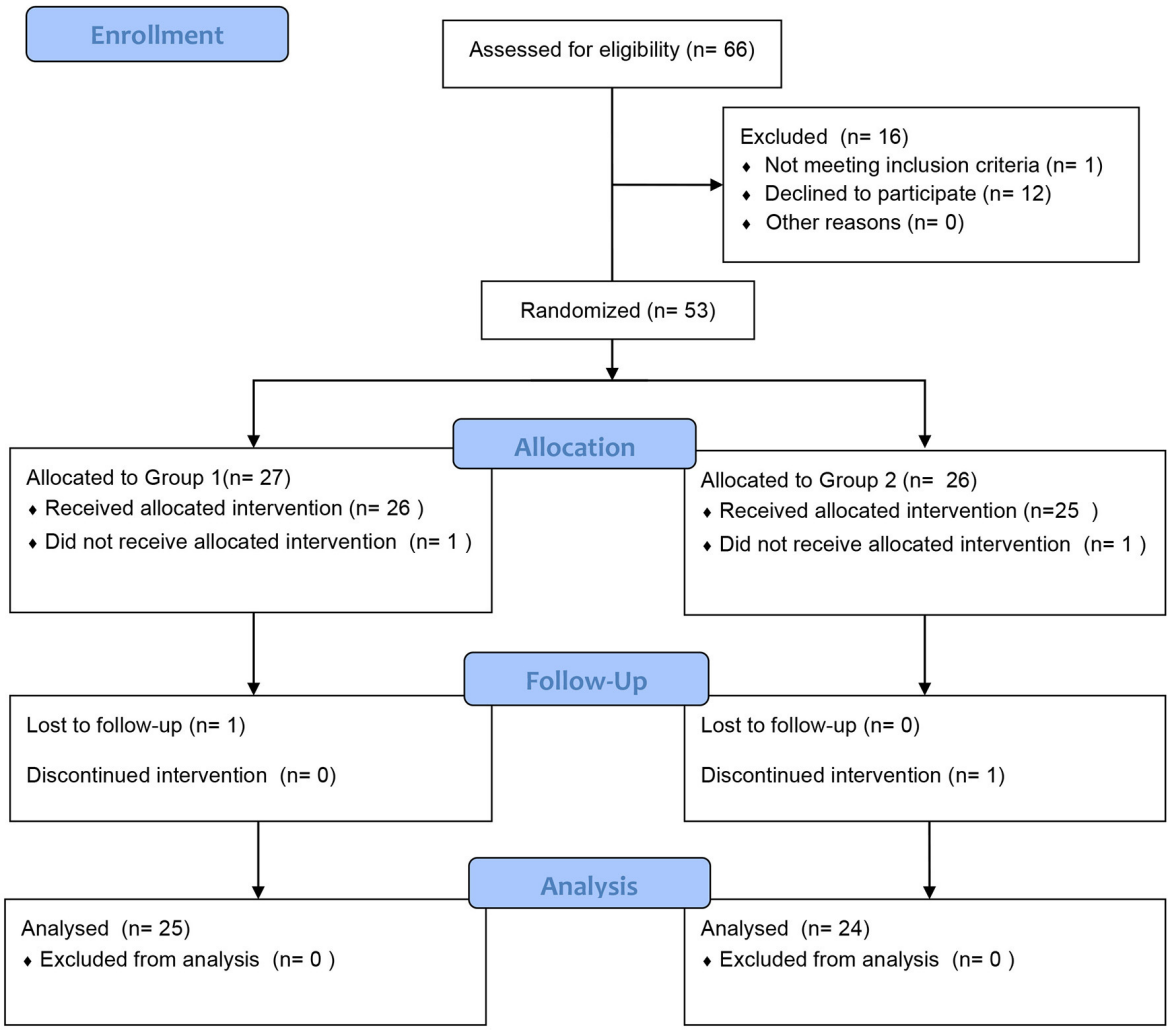
Patient diagram. In total, 66 subjects were recruited and randomly assigned to the two treatment groups to receive either acaricidal bait or placebo. Fifty children qualified and completed the first phase of the trial, and 49 children completed the entire trial.

In total, 66 patients with clinically proven AR and asthma were recruited. Fifty children qualified and completed the first phase of the trial, and 49 children completed the entire trial (Figure 2). The participants were randomly assigned to receive treatment with acaricidal bait and a placebo. The sample consisted of 31 males and 19 females. Table 1 summarizes the patient composition at baseline. There was no significant difference in the VAS, RCAT, RQLQ, and ACQ-5 scores or HDM antigen levels between Group 1 (first acaricidal bait and then placebo) and Group 2 (first placebo and then acaricidal bait) before the treatment (*P* > 0.05). All subjects were allergic to dust mites, 11 subjects were allergic to pet hair, and 6 subjects were allergic to mold.

**Table 1:** Comparison between Group 1 and Group 2 before the treatment.

**Variables**	**Group 1** **(** ***n*** **= 25)**	**Group 2** **(** ***n*** **= 25)**	***P*** **-Value**
Age (y), median (IQR)	6 (4)	6 (4)	0.271
Gender, *n* (%)			0.145
Male	13 (52%)	18 (72%)	
Female	12 (48%)	7 (28%)	
RCAT, median (IQR)	19 (5.5)	22 (6.5)	0.447
VAS, median (IQR)	18 (18.5)	25 (29)	0.497
RQLQ, median (IQR)	9 (9)	12 (13.5)	0.515
ACQ-5, median (IQR)	0.2 (1.1)	0.4 (0.9)	0.668
Der p2+Der f2 (μg/g), median (IQR)	3.209 (3.558)	2.217 (3.618)	0.432
HDM sIgE (IU/ml), median (IQR)	8.5(23.35)	15.1(23.2)	0.290

*IQR, interquartile range; RCAT, rhinitis control assessment test score; VAS, visual analog scale score of rhinitis symptoms; RQLQ, rhinoconjunctivitis quality-of-life questionnaire score; ACQ-5, asthma control questionnaire-5 score; Der p, Dermatophagoides pteronyssinus; Der f, D. farina; HDM sIgE, House dust mite specific immunoglobulin E*.

### Effect of Acaricide Bait in the Crossover Design

**Figure 3:**
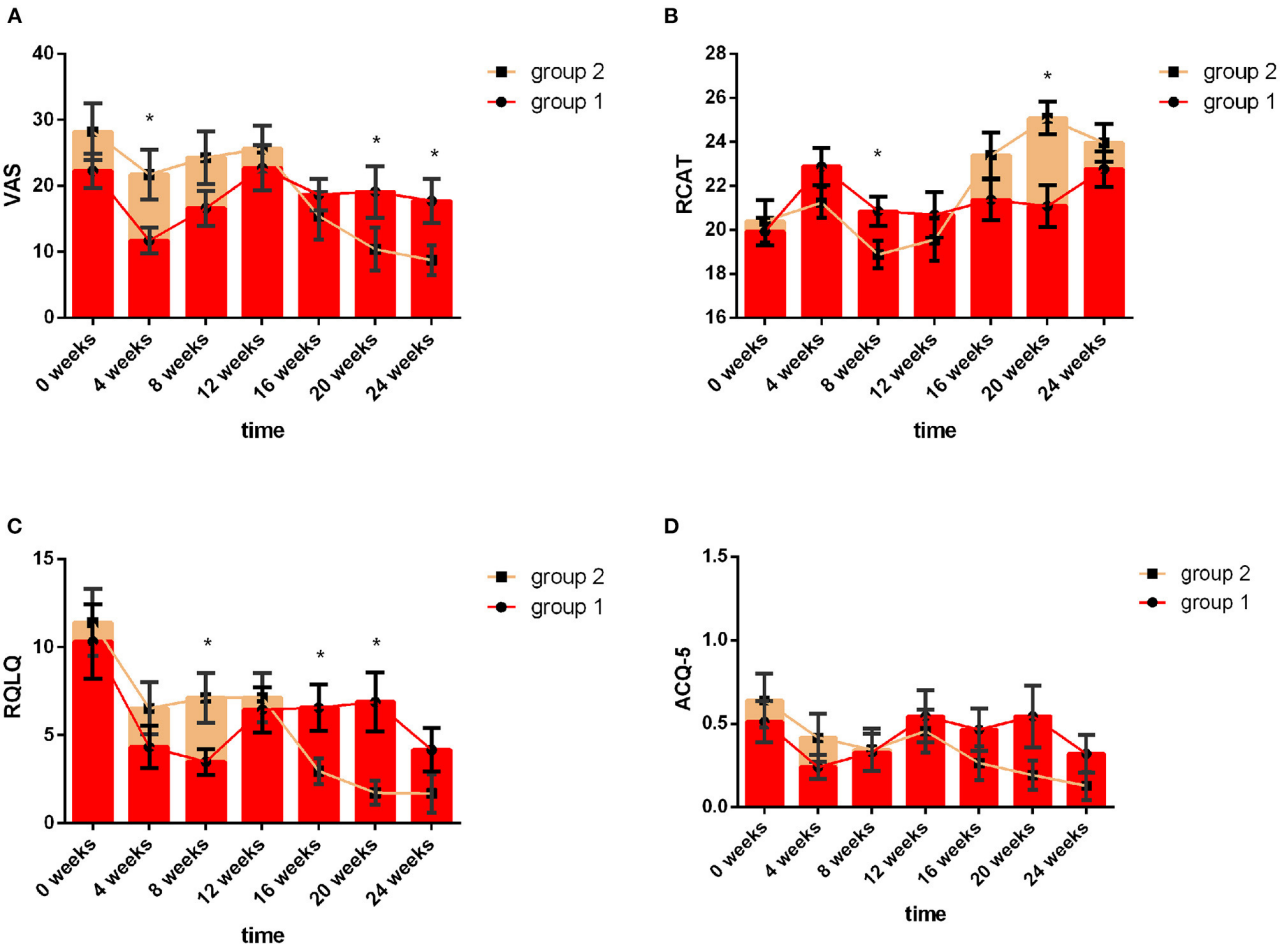
VAS, RCAT, RQLQ and ACQ-5 scores of the two groups at different follow-up visits. The red line indicates Group 1, and the yellow line indicates Group 2. Week 0 to Week 8 refer to the first intervention phase: Group 1: acaricidal bait, Group 2: placebo; Week 8 to Week 12 refer to the washout period; Week 12 to Week 20 refer to the second intervention phase: Group 1: placebo, Group 2: acaricidal bait. Changes in the VAS **(A)**, RCAT **(B)**, RQLQ **(C)**, and ACQ-5 **(D)** scores between the acaricidal bait and placebo groups at the same time points. Bars indicate the mean values, and brackets represent the standard error. *P < *0.05*.

Group 1 used acaricide bait from week 0 to week 8, and Group 2 used acaricide bait from week 12 to week 20. The mean values of the VAS, RCAT, RQLQ, and ACQ-5 scores in the two groups at the same treatment stage are shown in Figure 3. At baseline, there were no significant differences in the VAS, RCAT, RQLQ, or ACQ-5 scores between the two groups (*P* > 0.05). In week 4, the VAS scores of the children in the acaricide bait group were significantly lower than those of the children in the placebo group (*P* < 0.05). In week 8, the RCAT scores of the children who used acaricide bait were significantly higher than those of the children who used placebo (*P* < 0.05), and the RQLQ scores in the acaricide group were significantly lower than those in the placebo group (*P* < 0.05). After a washout period of 4 weeks, the VAS, RCAT and RQLQ scores in Group 2, i.e., cross-treatment with acaricidal bait in week 20, were also better than those of the children using placebo during the same period. Although the children who used acaricidal bait at the same time had lower ACQ-5 scores than the children in the placebo group during the same period, there was no significant difference. In summary, the research results based on the crossover design showed that the indoor use of acaricide bait improved rhinitis symptoms, rhinitis control and the quality of life of children with rhinitis, but the effect on asthma symptom control in the children was not significant.

### Acaricide Bait and Placebo Effects

**Figure 4:**
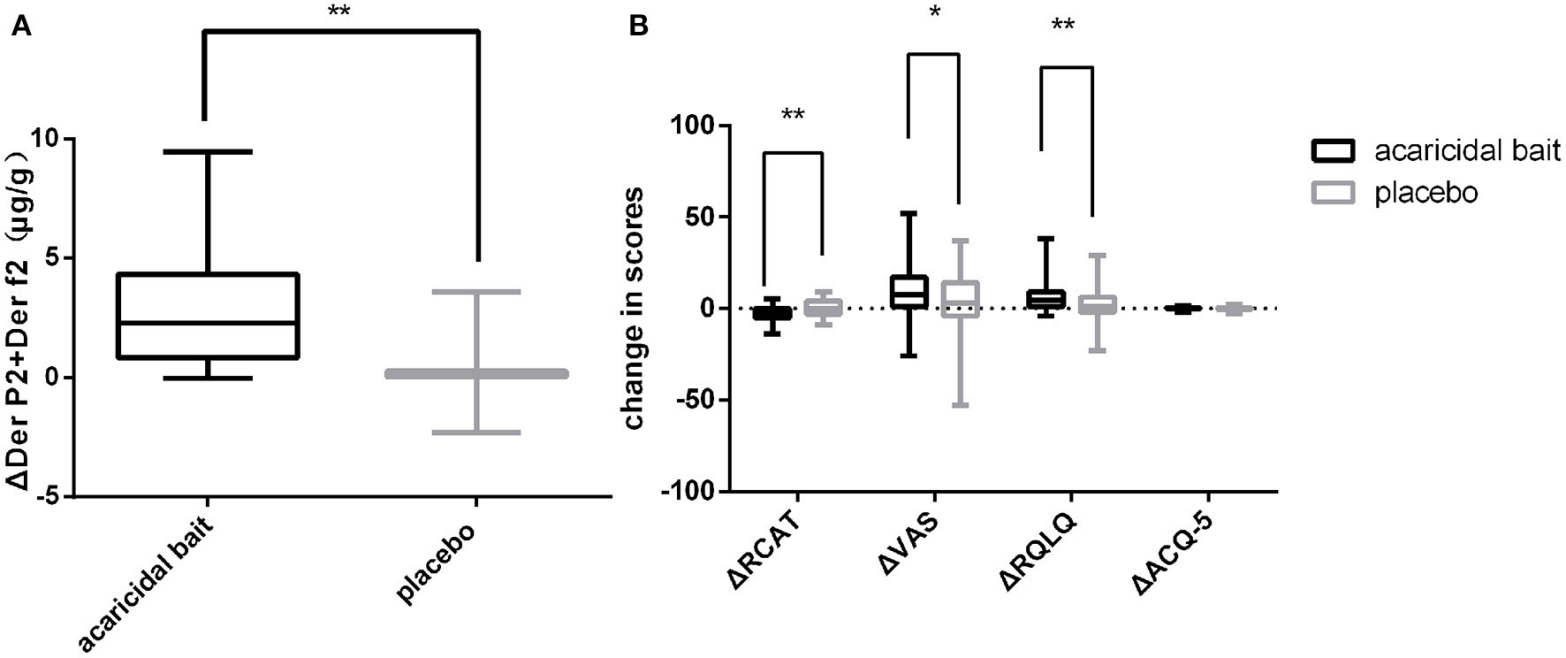
After 8 weeks of use, a comparison of ΔDer p2+Der f2 **(A)**, ΔRCAT, ΔVAS, ΔRQLQ and ΔACQ-5 **(B)** between the placebo and acaricidal bait groups. Boxes indicate the median, and whiskers represent the maximum value to minimum value. ΔDer p2+Der f2 = pretreatment dust mite antigen concentration subtracted from the posttreatment dust mite antigen concentration. ΔRCAT, ΔVAS, ΔRQLQ and ΔACQ-5 = pretreatment scores subtracted from the posttreatment scores. *P < 0.05, **P < 0.01.

As shown in Figure 4A, the decline in HDM antigens in the mattresses after using acaricidal bait was more obvious than that in the placebo group (*P* < 0.01). Similarly, the subjects in the acaricidal bait group showed statistically significant improvements in rhinitis symptoms and rhinitis quality of life scores compared with the children in the placebo group (*P* < 0.05) based on the VAS, RCAT and RQLQ scores for rhinitis (Figure 4B). However, the improvement in the control of asthma did not significantly differ between the two groups based on the ACQ-5 scores. The study was designed as a crossover trial, as shown in Tables 2, 3. After excluding individual differences between the selected children and the effects of the intervention measures, acaricidal bait still had a significant effect on improving the symptoms of rhinitis and reducing the number of dust mite antigens.

**Table 2:** Cross-analysis of the decrease in the VAS score.

**Source**	**F-value**	***P*** **-value**	**Partial η2**
Intercept	19.064	0.000	0.280
Stage	3.037	0.088	0.060
Patient	1.509	0.078	0.606
Treatment	6.388	0.015^*^	0.117

* *P < 0.05*.

**Table 3:** Cross-analysis of the decrease in Der p2+Der f2.

**Source**	**F-value**	***P*** **-value**	**Partial η2**
Intercept	83.183	0.000	0.629
Stage	1.330	0.425	0.517
Patient	0.773	0.814	0.441
Treatment	52.573	0.000^**^	0.523

** *P < 0.01*.

### Factors Affecting Asthma Control

An ACQ-5 score greater than or equal to 0.75 indicates that asthma is incompletely controlled. A multivariate model adjusted for gender, age, acaricidal bait, and INCs revealed that the use of ICS (OR 0.309; 95% CI 0.116–0.82; *P* < 0.05) and a good rhinitis control status (OR 0.082; 95% CI 0.027–0.242; *P* < 0.001) were independently associated with good asthma control (Table 4; area under the curve, 0.804).

**Table 4:** Multivariate logistic regression analysis with an ACQ-5 score ≥ 0.75 as the outcome.

**Characteristics**	**OR (95% CI)**	***P*** **-value**
Gender	2.071 (0.838–5.116)	0.115
Age	1.328 (0.549–3.213)	0.528
Treatment	0.707 (0.301–1.661)	0.427
Intranasal corticosteroids	2.371 (0.972–5.786)	0.058
Inhaled corticosteroids^*^	0.309 (0.116–0.82)	0.018
RCAT^*^	0.082 (0.027–0.242)	0

*RCAT, rhinitis control assessment test score. AUC (area under the curve) = 0.804, n = 200*.

* *Independent predictors of incomplete asthma control as assessed by the ACQ-5*.

### Adverse Events

Of the 66 children recruited, 16 children withdrew from the study during the first phase because they did not meet the selection criteria or were lost to follow-up. After completion of the first phase of the study, the parent of one child reported that the child's rhinitis symptoms had not improved and withdrew the child from the second phase of the study. After unblinding, the child was identified as being in the placebo group. The child's symptoms improved after outpatient treatment. Throughout the entire research process, no serious adverse reactions were reported.

## Discussion

Dust mites are one of the strongest allergens found so far (20). They are an important factor in causing indoor allergic diseases. The allergic diseases caused by them mainly include: Allergic asthma, allergic rhinitis, allergic dermatitis, allergic eczema, etc. The present study evaluated changes in the concentrations of HDM antigens in mattresses and the symptoms of children with rhinitis and asthma after the use of acaricide bait. The results showed that acaricide bait significantly reduced the concentrations of HDM antigens in the mattresses and improved rhinitis symptoms, rhinitis control and quality of life. These results indicate that using acaricide bait is an effective method to control children's AR symptoms. However, some previous studies yielded different results. Previous studies (21–26) investigated the effect of acaricides as a single intervention to eradicate HDM allergens in mattresses, carpets and interior decorations. The results demonstrated that acaricides reduced the burden of HDM antigens, but the results of rhinitis or improvement in asthma symptoms were unclear. This difference may be related to the fact that ordinary acaricides cannot penetrate carpets or mattresses and can only kill live mites on the surface, which does not effectively prevent allergies. Differently, acaricide bait actively attracts mites into the package, and the HDMs die after eating the acaricide in the package such that HDM allergens do not diffuse into the air.

In addition, we also evaluated the improvement of asthma children's symptoms after using acaricide baits due to that allergic rhinitis and asthma are common comorbid diseases. The results showed that the acaricide bait did not significantly improve asthma symptoms. Similar to the results of the present study, several systematic reviews and meta-analyses concluded that most studies evaluating the impact of a single intervention or multiple interventions failed to show improvements in the main asthma outcome (27–29). However, a recent randomized controlled study involving children (30) showed some different results based on the above conclusions. This randomized study suggested that reducing exposure to HDM allergens was effective in reducing emergency hospital attendance for severe asthma exacerbations after using HDM–impermeable bedding because significantly fewer children in the active group than in the placebo group were admitted to the hospital due to an exacerbation (36 [29.3%] of 123 vs. 49 [41.5%] of 118; *P* = 0.047) at 12 months. Acaricide bait did not improve the asthma control status in the present trial. The following reasons for the differences in the results are suggested. (1) Some previous studies showed that the threshold of 10 μg/g HDM allergens was related to asthma symptoms (31). It is possible that the baseline level of HDM allergens in the present study was too low, and, consequently, the reduction in allergen exposure could only be moderate. Therefore, the control of asthma was not significantly improved after using acaricidal bait. (2) Another potential problem is that the present trial did not conduct stratification according to asthma severity. In the study, 68% of the children with asthma based on the ACQ-5 scores were in a well-controlled state; therefore, these children's asthma symptoms may not significantly improve after reducing HDM exposure.

However, the existence of AR is certainly related to poor asthma control. A recent study reported that the persistence and severity of AR were associated with the level of asthma control in patients with AR and asthma (32). Similarly, the present study revealed that after adjusting for potential confounding factors, good control of rhinitis and the use of ICS were beneficial for the control of asthma symptoms. This finding is similar to the abovementioned research conclusions and highlights the importance of the joint management of airways.

At the same time, we also notice that there are some limitations of this study. First, the sample size of the study was small, and the present study was mainly concentrated in autumn and winter, while spring is the main season for HDM reproduction in southern China; thus, the effect of acaricide bait on reducing HDM exposure may be underestimated. Second, not all included patients in this study were allergic to HDMs alone (all subjects were allergic to dust mites, 11 subjects were allergic to pet hair, and 6 subjects were allergic to mold); however, according to an epidemiological study (33), HDM is the dominant allergen in AR patients in southern China. Those who were allergic to pet hair did not have pets. Therefore, HDM may be the main allergen in these patients. Finally, the random grouping, double-blind, crossover test method adopted in this study reduces the influence of selection bias, measurement bias, and other errors on the test results. Each subject received two schemes successively, enabling before-and-after comparisons and eliminating individual differences while permitting intergroup comparison. However, due to the long period of this study, problems, such as loss to follow-up, withdrawal, and decline in compliance, may easily occur. Ensuring that each patient is in the same condition during each phase of treatment is difficult.

In general, the present study showed the effectiveness of acaricide bait in reducing HDM antigens and improving rhinitis symptoms, rhinitis control, and quality of life and the importance of cotreatments for rhinitis and asthma. Due to its convenience, safety and effectiveness, the promotion of acaricide bait for use in family residences has broad prospects and may become an effective means for the management and treatment of rhinitis and asthma in the future.

## Data Availability Statement

The original contributions presented in the study are included in the article/supplementary material, further inquiries can be directed to the corresponding author/s.

## Ethics Statement

The studies involving human participants were reviewed and approved by Institutional Review Board of Shanghai Children's Medical Center Affiliated with Shanghai Jiaotong University of Medicine. Written informed consent to participate in this study was provided by the participants' legal guardian/next of kin.

## Author Contributions

YY obtained funding. MC, YW, and JZ designed the study. JC, LL, and JW are members of the data monitoring committee responsible for monitoring the recruitment of participants, compliance with the protocol and data quality, and monitoring for serious adverse events and other safety questions. LZ and MT analysed the data. All authors contributed to drafting, revising and editing the article, gave final approval of the version to be published, and agree to be accountable for all aspects of the work.

## Funding

This study was supported by the Scientific Research Project of Shanghai Pudong New Area Health Committee (No. PW2017E-1) and Scientific Research Projects of Shanghai Science and Technology Commission (No. 19441909000). These funding bodies played no role in the study design, collection, analysis, and interpretation of the data, or writing of the manuscript.

## Conflict of Interest

The authors declare that the research was conducted in the absence of any commercial or financial relationships that could be construed as a potential conflict of interest.

## Publisher's Note

All claims expressed in this article are solely those of the authors and do not necessarily represent those of their affiliated organizations, or those of the publisher, the editors and the reviewers. Any product that may be evaluated in this article, or claim that may be made by its manufacturer, is not guaranteed or endorsed by the publisher.

## Abbreviations

AR: Allergic rhinitis
HDMs: House dust mites
VAS: Visual analog scale
RCAT: Rhinitis Control Assessment Test
RQLQ: Rhinoconjunctivitis Quality of Life Questionnaire
ACQ-5: Asthma Control Questionnaire-5
INCs: Intranasal corticosteroids
ICS: Inhaled corticosteroids
Der p: *Dermatophagoides pteronyssinus*
Der f: *D. farina*.
